# How the Management of Children With Congenital Central Hypoventilation Syndrome Has Changed Over Time: Two Decades of Experience From an Italian Center

**DOI:** 10.3389/fped.2021.648927

**Published:** 2021-03-29

**Authors:** Federica Porcaro, Maria Giovanna Paglietti, Claudio Cherchi, Alessandra Schiavino, Maria Beatrice Chiarini Testa, Renato Cutrera

**Affiliations:** Pediatric Pulmonology & Respiratory Intermediate Care Unit, Sleep, and Long-Term Ventilation Unit, Academic Department of Pediatrics and Genetic Research Area, Bambino Gesù Children's Hospital, IRCCS, Rome, Italy

**Keywords:** congenital central hypoventilation syndrome, PHOX2B gene, genotype/phenotype correlation, ventilatory mode, children

## Abstract

**Background:** Congenital central hypoventilation syndrome (CCHS) is a rare disorder whose clinical phenotype is closely related to genotype.

**Methods:** A retrospective analysis has been conducted on 22 patients with CCHS, who were referred to the Pediatric Pulmonology and Respiratory Intermediate Care Unit of Bambino Gesù Children's Hospital (Italy) for a multidisciplinary follow-up program between 2000 and 2020.

**Results:** Apnea and cyanosis were the most frequent symptoms at onset (91%). Overall, 59% of patients required tracheostomy and invasive mechanical ventilation (IMV) in the first months of life. Thirty-two percent of patients had Hirschsprung disease (HSCR) that was associated with longer polyalanine repetitions or non-polyalanine repeat expansion mutations (NPARMs). Polyalanine repeat expansion mutations (PARMs) were more frequent and two novel NPARMs (c.780dupT and C.225–256delCT) were described in 14% of patients. Focal epilepsy was first described in 14% of patients and neurocognitive and neuromotor impairment involved 27% and 23% of children, respectively. Symptoms due to autonomic nervous system dysfunction/dysregulation (ANSD)—including strabismus (27%), dysphagia (27%), abnormal heart rhythm (10%), breath-holding spells (9%), and recurrent seizures due to hypoglycemia (9%)—were associated with an increased number of polyalanine repetitions of exon 3 or NPARMs of PHOX2B gene. Overall, the number of patients with moderate to severe phenotype initially treated with non-invasive ventilation (NIV) increased over time, and the decannulation program was concluded with 3 patients who started with IMV.

**Conclusions:** Our study confirms that more severe phenotypes of CCHS are related to the number of polyalanine repetitions or to NPARMs. Although invasive ventilation is often required by patients with severe genotype/phenotype, gradual acquisition of specific skills in the management of patients with CCHS and technological improvements in mechanical ventilation allowed us to improve our therapeutic approach in this population.

## Introduction

Congenital central hypoventilation syndrome (CCHS) is a rare life-threatening disorder characterized by autonomic dysregulation and alveolar hypoventilation, requiring lifelong ventilatory assistance. Affected subjects are unable to perceive and respond to hypercarbia with increased ventilation and arousal during sleep (1). Even though hypoventilation is accentuated during sleep, especially during the non-rapid eye movement (NREM) phase (2), more severely affected individuals exhibit it even when they are awake (3).

PHOX2B gene mutations are considered to be responsible for most cases of CCHS (3), and over 1,000 cases of PHOX2B mutation-confirmed CCHS are reported by different laboratories in 2009 (3).

PHOX2B gene, encoding a transcription factor involved in autonomic system development, has a 20-polyalanine sequence in exon 3 (4).

Over 90% of CCHS cases are heterozygous for polyalanine repeat expansion mutations (PARMs) in the exon 3 of PHOX2B gene producing genotypes of 20/24 to 20/33, that often arise *de novo* (5).

The remaining patients with classic CCHS are heterozygous for non-sense, missense, or frameshift mutations (NPARMs) located on exons, 1, 2, or 3 of the PHOX2B gene. A recent study shows the different genetic mutations related to MYO1H gene as the cause of a rare recessive form of CCHS (6).

A genotype–phenotype correlation is well-described and the severity of the symptoms—including continuous ventilator dependence, heart rhythm disorders, association with Hirschsprung's disease (HSCR), and neural crest tumors—is closely related to NPARMs and to the number of alanine repetitions among subjects with PARMs (7–10).

Moreover, the appearance of symptoms of the autonomic nervous system dysfunction/dysregulation (ANSD) listed in Table 1 is linked to NPARMs and more repetitions of PARMs that contribute to the severity of the phenotype (4).

**Table 1:** Symptoms of the Autonomic Nervous System dysregulation described in CCHS patients.

**LIST OF SYMPTOMS**
Diminished pupillary light response
Esophageal dysmotility
Breath-holding spells
Low basal body temperature
Profuse sweating
Lack of physiologic response to the stressors

The symptoms of CCHS typically occur in the first months of life (3), even though a milder later-onset (LO-CCHS) presentation is described in toddlers, children, and adults with a history of central hypoventilation and/or cyanosis or seizures arisen after anesthetics or administration of CNS depressants, recent severe lung infection, or the treatment of obstructive sleep apnea (3).

As CCHS does not resolve spontaneously, chronic ventilator support *via* tracheostomy, non-invasive ventilation (11–13), negative-pressure ventilation, or diaphragm pacing (14, 15) are considered as possible therapeutic options for these patients.

The aims of our study are: (1) to describe the genotypes and the clinical findings of patients affected with CCHS followed by a third level of care in a pediatric hospital and (2) to highlight the changing strategy in the management of these patients over time.

## Materials and Methods

Data of 22 patients affected by CCHS has been collected retrospectively. Enrolled patients were referred to the Pediatric Pulmonology and Respiratory Intermediate Care Unit of Bambino Gesù Children's Hospital (Rome, Italy) for a multidisciplinary follow-up program between 2000 and 2020. Our Respiratory Unit is a reference center for children affected with acute or chronic respiratory failure due to neurologic or neuromuscular impairment and by congenital or acquired lung diseases. Our hospital is the reference point for the Italian pediatric population, especially from central and southern regions.

Once the nurses, physiotherapists, and doctors of our team obtained the necessary expertise on CCHS, 14 patients have been followed in our Pediatric Intermediate Care Unit (PICU) since the onset of symptoms. The remaining 8 patients were initially treated by the staff of the Intensive Care Unit (ICU) of our hospital and then referred to our Unit from 2010.

Demographic data, medical histories, and results of the diagnostic testing were obtained from the review of the electronic medical record with the approval from the Institutional Review Board.

The following criteria were considered for the diagnosis of CCHS: (i) persistent central alveolar hypoventilation (PaCO_2_ > 60 mm Hg) during sleep, detected by polysomnography, while the patient spontaneously breathes the room air, (ii) lack of ventilator responses to hypercarbia, and (iii) absence of primary lung, neuromuscular, or heart diseases (1).

When central hypoventilation and hypercarbia were confirmed on nocturnal polysomnography and transcutaneous capnography, blood samples of the probands and their parents were collected to perform genetic investigations, after obtaining written informed consent.

Capillary Sanger amplification and sequencing techniques of coding regions and exon–intron junctions of the PHOX2B gene were used to confirm the genetic diagnosis (16).

**Figure 1:**
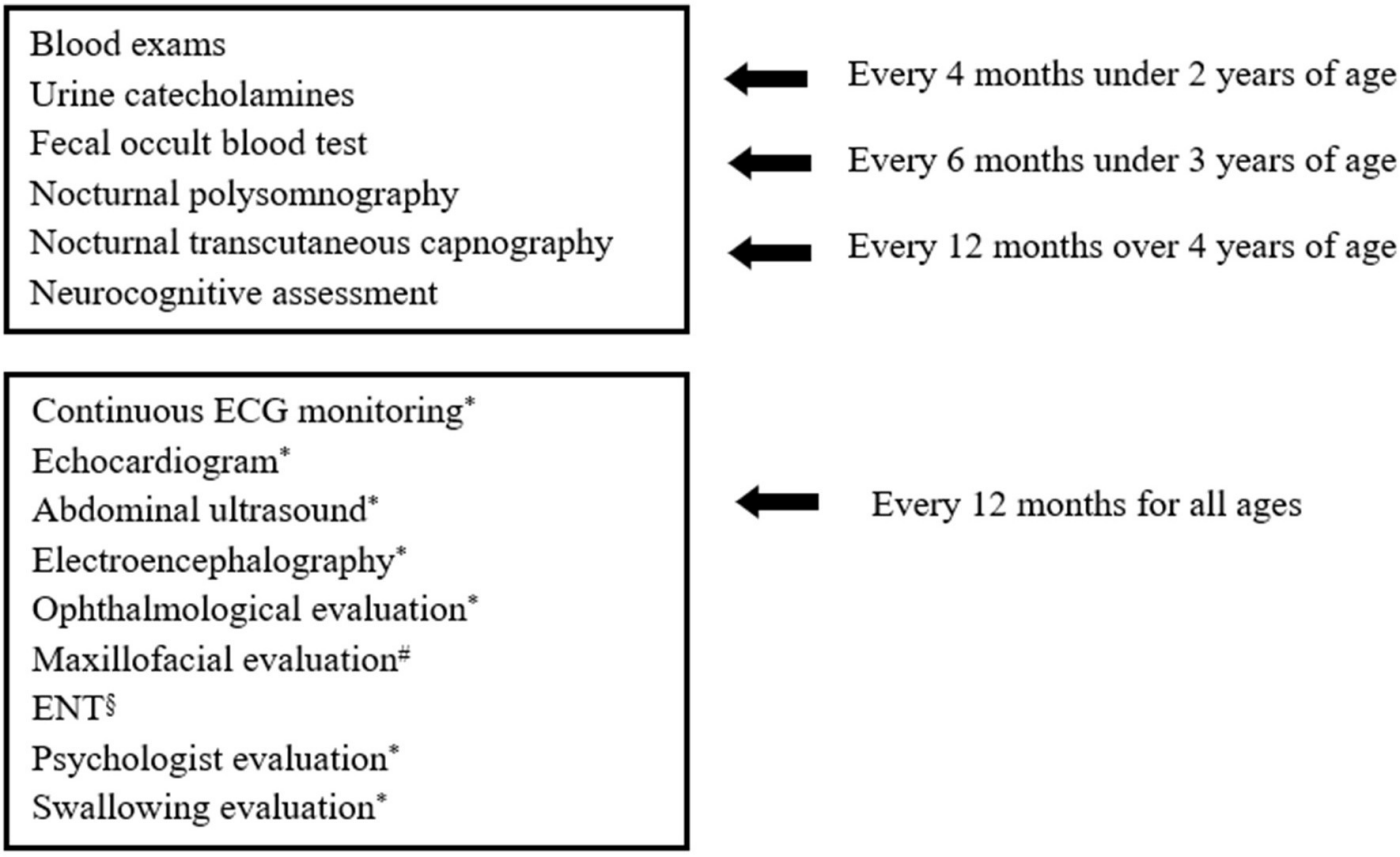
Investigations included in Bambino Gesù Children's Hospital follow-up program for patients with CCHS. ^#^in case of NIV. ^§^in case of tracheostomy. *evaluations carried out annually or as needed.

Once the diagnosis was made, patients were included in a follow-up program which has been changed over time and whose current version is summarized in Figure 1. Patients with a history of delayed meconium elimination and chronic constipation were highly suspected for HSCR; hence, a rectal suction biopsy was performed, and histopathological analysis was carried out. HSCR was confirmed by the absence of ganglion cells in the myenteric and submucosal plexuses of the distal intestine, resulting in the lack of peristalsis and functional intestinal obstruction (17). All the collected data are expressed as numbers, means ± SD, and percentages.

## Results

Twenty-two Caucasian patients with CCHS, who were followed in the last 20 years by our Respiratory Unit, were included in the study. The median age at the time of diagnosis was 2 months (interquartile range, IQR 1–3 months) for 20 children. The diagnosis was occasionally made at 35 and 38 years in two adult female patients, whose CCHS was retrospectively confirmed after the detection of genetic mutation in their children. Ten patients (45%) were male. Mean gestational age was 37.5 weeks (±2.6 SD, range 31–41), and the mean neonatal weight was 2,982.72 g (± 698.43 SD, range 1,350–4,100).

Apnea and cyanosis were the most frequent symptoms occurring in the first few days of life in 91% of patients with suspicion for an increase in CCHS.

All patients underwent nocturnal polysomnography and transcutaneous capnography, and 21 cases showed central hypoventilation and hypercapnia. Only one adult female patient with a past medical history of surgically treated HSCR had a normal sleep study and still did not require ventilation. In addition, all patients were screened for other associated conditions, including abnormalities of the central nervous system (CNS), neurocognitive impairment, behavioral disorders, heart diseases, and neural crest tumors.

The definitive diagnosis was made through Sanger amplification and sequencing techniques of coding regions and exon–intron junctions of the PHOX2B gene.

Molecular analysis demonstrated common PARMs (20/25, 20/26, and 20/27) in 19/22 cases (86%) and NPARMs in 3/22 cases (14%). Results of the genetic analysis are presented in Table 2.

**Table 2:** PHOX2B gene mutations and ventilation mode for 22 of our patients.

**Number**	**Type of mutations**	**Mutations**	**Ventilation**
N1	PARMs	20 > 26	IMV
N2	PARMs	20 > 26	IMV
N3	PARMs	20 > 26	IMV/NIV
N4	PARMs	20 > 25	NIV
N5	PARMs	20 > 26	IMV
N6	PARMs	20 > 25	NIV
N7	Frameshift	C.225–256delCT	none
N8	PARMs	20 > 26	NIV
N9	PARMs	20 > 26	IMV
N10	PARMs	20 > 26	NIV
N11	PARMs	20 > 25	NIV
N12	Frameshift	c.780dupT^*^	IMV
N13	PARMs	20 > 27	IMV
N14	PARMs	20 > 26	IMV/NIV
N15	PARMs	20 > 25	IMV
N16	PARMs	20 > 26	NIV
N17	PARMs	20 > 27	IMV
N18	PARMs	20 > 26	IMV/NIV
N19	PARMs	20 > 27	IMV
N20	PARMs	20 > 27	IMV
N21	PARMs	20 > 25	NIV
N22	Frameshift	C.225–256delCT	NIV

* *Only patients with c.780dupT mutation and HIE showed high dependence on ventilation (22 h/day). PARMs; Polyalanine repeat expansion mutations*.

### Respiratory Manifestations

**Figure 2:**
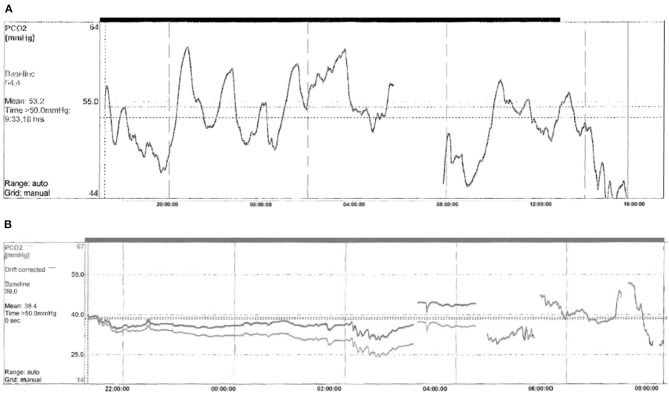
Example of capnography at baseline **(A)** and after starting NIV **(B)** in a newly diagnosed infant with CCHS. **(A)** Capnography made in spontaneous breathing. Monitoring time 10.18 h mean TcPCO2 53.2 mmHg, maximum TcPCO2 61.2 mmHg, minimum TcPCO2 46 mmHg, and time % spent with TcPCO2 > 50 mmHg 84%. **(B)** Capnography made on NIV. Monitoring time 10.24 h mean TcPCO2 38.4 mmHg, maximum TcPCO2 47.3 mmHg, minimum TcPCO2 34.2 mmHg, and time % spent with TcPCO2 > 50 mmHg 0.0%.

**Figure 3:**
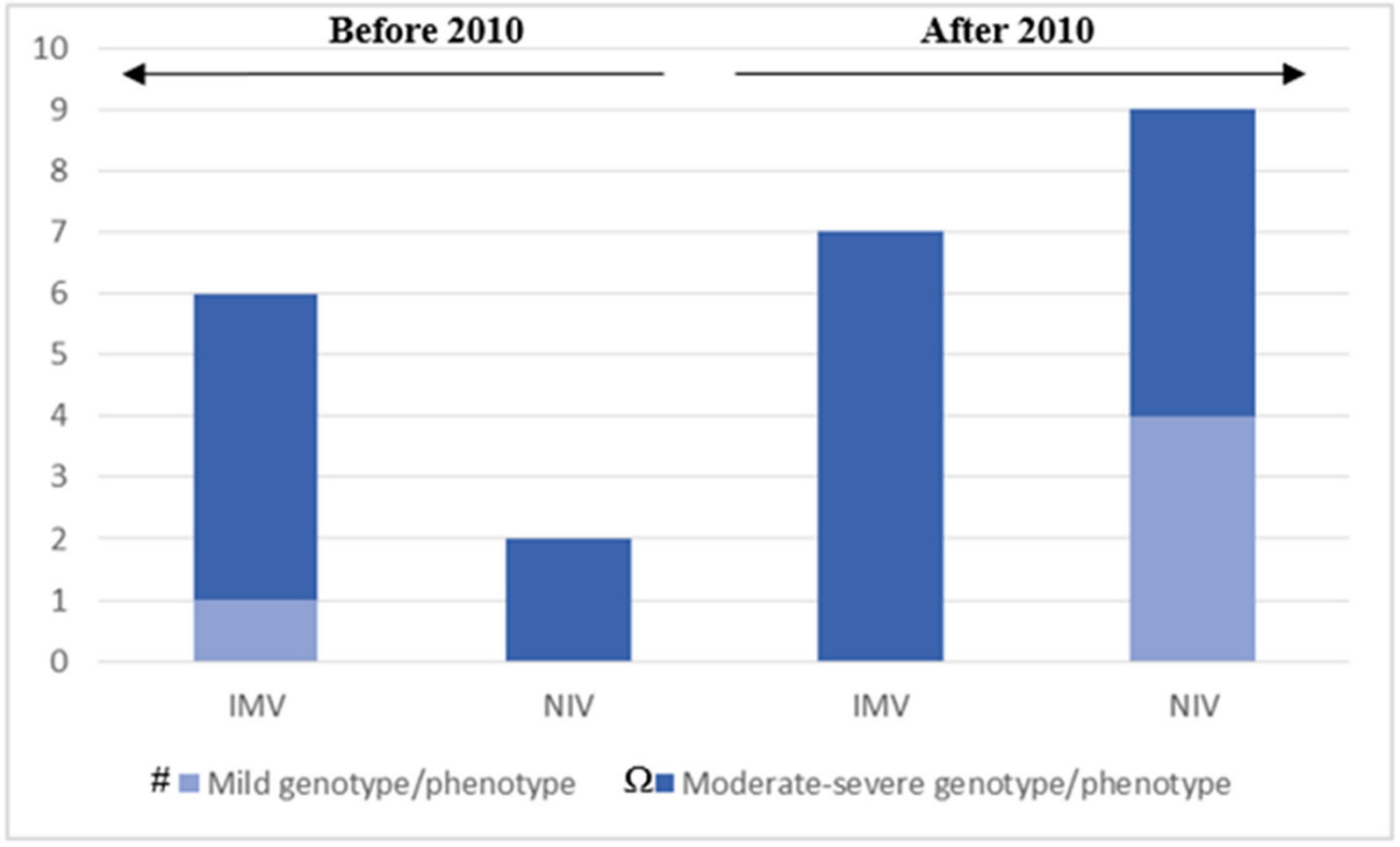
The chart represents our clinical approach in different decades: after 2010, IMV has been reserved for patients with moderate to severe genotype/phenotype; NIV has been applied as the first choice for ventilator support in patients with mild and moderate to severe genotype/phenotype. # Mild genotype/phenotype: PARMs 20/25. Ω Moderate-severe genotype/phenotype: PARMs 20/26, 20/27; NPARMs C.225_256delCT, c.780dupT. One adult female patient that still doesn't need ventilation is not included. One adult female patient that started NIV in adult age is included in the fourth column. The 3 patients with moderate-severe genotype/phenotype that carried out the decannulation on program are also included in the fourth column.

Focusing on ventilation impairment, 13/22 (59%) patients underwent tracheostomy at the mean age of 3.92 ± 3.19 months (range 1–13), and needed invasive mechanical ventilation (IMV). The duration of IMV has been reduced over time, according to the age of the patients and individual sleep–wake rhythm, even though the child with c.780dupT mutation and hypoxic ischemic encephalopathy (HIE) still has a 22 h/day ventilation dependency. Children starting directly on IMV were usually patients with more severe genotype (20/26, 20/27, or NPARMs), and sometimes additional neurological impairment due to neonatal hypoxia. Only one male patient with mild genotype (20/25) and born in 1989, started directly on IMV because of the lack of non-invasive ventilation (NIV) experience within the ICU team (**Figure 3**). Ventilation has been updated on the basis of CO_2_ monitoring, obtaining satisfactory average CO_2_ levels for all tracheostomized patients (mean CO_2_ value of 39.5 mm Hg, maximum CO_2_ value of 49.6 mm Hg, 4% of the time spent over 50 mm Hg of CO_2_). Three of the tracheostomized children with 20/26 genotype started weaning from IMV and training to NIV at the mean age of 106.25 months (± 40.7 SD). The switch to NIV has been considered only in patients fulfilling all the following criteria: (1) normal consciousness state of the patient, (2) intact cough reflex and adequate managing respiratory secretions, (3) need of suction ≤1 time/day, (4) daytime tolerated tracheostomy capping, (5) IMV dependency only during sleep, (6) integrity of the upper and lower airways on bronchoscopy assessment, (7) no history of recurrent respiratory exacerbations requiring hospitalization in the previous 12 months, and (8) motivation from patient and his family. Finally, decannulation occurred at a mean age of 120.2 months (±42.3 SD) (18) with a good control of average CO_2_ levels on subsequent visits (mean CO_2_ value of 42.5 mm Hg, maximum CO_2_ value of 55.2 mm Hg, 4% of the time spent over 50 mm Hg of CO_2_). Seven children (32%) started directly with NIV at the mean age of 24.57 months (±24.18 SD, range 1–60). Two patients with 20/26 genotype started NIV in the ICU setting; meanwhile, five children with genotype ranging from mild to severe (20/25, 20/26, and C.225_256delCT) started NIV in the PICU setting after 2010. Due to the young age and the amount of sleep time, adaptation to ventilation was particularly difficult. Nevertheless, good average CO_2_ levels have been obtained in this group of patients (mean CO_2_ value 41.9 mm Hg, maximum CO_2_ value of 49.3 mm Hg, 14% of the time spent over 50 mm Hg of CO_2_) after several and close evaluation of ventilation setting (Figure 2). Only one female patient started NIV when the diagnosis was made in adult age.

The acquisition and maintenance of specific skills in the field of CCHS and NIV by the PICU team over time, allowed NIV to become the preferred initial treatment also for the CCHS-affected children with severe genotype/phenotype (Figure 3), in the last 10 years.

### Gastrointestinal Symptoms

Seven patients (32%) reported a history of bowel obstruction suggesting HSCR, which was histologically confirmed and surgically corrected. Surgery was made in the first 3 months of life in five cases with long (4 patients) and short (1 patient) segment HSRC. Two adult female patients having nuanced intestinal symptoms due to a short segment of HSCR underwent surgery belatedly in the 2nd year of life.

### Neurologic and Psychological Manifestations

Focal epilepsy was detected in 3/22 children (14%) and specific therapy was necessary to control the neurologic symptoms. Neurocognitive and movement impairment was detected, respectively, in 6/22 (27%) and 5/22 (23%) cases; it is possible that neonatal hypoxia contributed to neurological impairment in two cases as confirmed by the detection of ischemic lesions on brain MRI. Six patients (27%) had ocular motility impairment as convergent strabismus. Lastly, behavioral disorders were detected in 5/22 cases (23%) and dysphagia was observed in 6/22 patients (27%) requiring gastrostomy.

### Cardiovascular Manifestations

Six patients (27%) had heart abnormalities, such as tricuspidal insufficiency (50%), interatrial defect (33%), and patent ductus arteriosus (17%), and one child had abnormal heart rhythm (atrioventricular block), requiring pacemaker implantation.

### Autonomic Disturbances and Malignancies

Only two patients (9%) had breath-holding spells and recurrent seizures due to hypoglycemia. Instead, a neural crest tumor was recorded in one child in the 2nd year of life.

The clinical presentations of the 22 patients are summarized in Table 3. As shown in Tables 2, 3, broad genotype–phenotype correlations can be made, such as mutations with a greater number of polyalanine repeats or NPARMs are more likely to have more severe disease (need of IMV rather than NIV, severity of ventilation dependence, associated ANS, gastrointestinal, neurologic and cardiovascular symptoms, behavioral disorders, and rarely, the development of neural crest tumors).

**Table 3:** Genotype and phenotype features of our patients with CCHS.

**Number**	**Mutations**	**H&H**	**HSCR**	**HA**	**E**	**S**	**NMi**	**D**	**BHS**	**H&S**
N1	20 > 26	X	X							
N2	20 > 26	X	X	X						
N3	20 > 26	X	X			X				
N4	20 > 25	X								
N5	20 > 26	X				X		X		
N6	20 > 25	X			X		X			
N7	C.225_256delCT		X							
N8	20 > 26	X		X						
N9	20 > 26	X	X			X	X	X	X	
N10	20 > 26	X				X				
N11	20 > 25	X								
N12	c.780dupT	X	X			X	X	X	X	X
N13	20 > 27	X				X	X	X		
N14	20 > 26	X								
N15	20 > 25	X		X						
N16	20 > 26	X			X		X			
N17	20 > 27	X		X	X		X			
N18	20 > 26	X								
N19	20 > 27	X						X		X
N20	20 > 27	X		X				X		
N21	20 > 25	X								
N22	C.225_256delCT	X	X	X						

*H&H, hypoventilation & hypercapnia; HSCR, Hirschsprung's disease; HA, heart anomalies; E, epilepsy; S, strabismus; NMi, neuromotor impairment; D, dysphagia; BHS, breath-holding spells; H&S, hypoglycemia & seizure*.

## Discussion

The study describes the clinical and genetic findings of patients with CCHS, whose management—updated over time—allowed for the development of a well-defined follow-up program in the largest Italian pediatric hospital.

In accordance with the available scientific data (5), the typical symptoms of hypoventilation occurred in the first months of life in most patients, regardless of the type of the underlying gene mutation. Only two asymptomatic female patients—having a history of apparent isolated HSCR and seizures risen after general anesthetics administration—received the diagnosis after confirmation of the disease in their offspring.

As described in the literature (5), the vast majority of individuals were heterozygous for a PARMs involving the second polyalanine repeat sequence in exon 3 of PHOX2B. Instead, reported NPARMs were frameshift mutations on exon 3 of PHOX2B gene (C.225_256delCT, c.780dupT). Although the mutation C.225–256delCT has been detected in a woman and her son, the corresponding phenotype had surprisingly a variable expressiveness, as it was severe in the son. This observation suggests that somatic mosaicism or unknown environmental cofactors could influence the expression of the phenotype (3).

It is well-known that the severity of phenotype in CCHS is related to the presence of NPARMs and the number of alanine repeats among subjects with PARMs (19).

In our sample, the need for tracheostomy and invasive mechanical ventilation was dictated not only by the conditions of critical patients requiring the expertise of the ICU staff but also by management habits in 6 children born before 2010. Except for one child with mild genotype (20/25), severe ventilation impairment, and requiring tracheostomy, IMV was reported in 13 patients with more PARMs (20/26, 20/27) or NPARMs (c.780dupT) (Table 2). It is probable that the brain damage due to HIE contributed to worsening the ventilation impairment in two cases, who underwent IMV. Among tracheostomized patients, there were no differences in the age of the tracheostomy and starting of IMV, regardless of the genotype. All tracheostomized patients were ventilated during nighttime sleep and only the patient with c.780dupT mutation and HIE showed more ventilation dependency (about 22 h a day).

The progressive acquisition of non-invasive ventilator skills and the construction of a new and dedicated PICU setting allowed us to manage initially 8 patients with CCHS who had mild or moderate phenotype with NIV as well as to start weaning from IMV in three motivated patients (Table 2). Since the long-term use of nasal masks for NIV leads to the appearance of complications, such as pressure skin injury and midface hypoplasia, we started a close monitoring, together with maxillofacial surgeons and adopted a pro-active approach that included: (1) the correct choice of mask interface, (2) the regular use of protective skin coverings (hydrocolloids, foam pads, silicone, and gel) on areas with the highest contact pressure, and (3) regular interface rotation. No patient required maxillofacial interventions, thanks to these measures.

Although it is known that NPARMs are associated with a more risk of intestinal dysganglionosis (8), HSCR involved 32% of our entire sample, and especially 43% of patients had NPARM genotype and 57% had PARMs (20/26).

Three of our patients developed focal epilepsy during the follow-up period. Actually, no data are available on epilepsy and its association with CCHS. As each patient had a different genotype (20/25, 20/26, and 20/27), no correlation between epilepsy and genotype was finally seen. It is probable that prolonged hypoxia in patients receiving late diagnosis of CCHS contributed to neural damage and the development of abnormal brain electrical activity. It is also known that PHOX2B mutation-confirmed CCHS confers the risk for adverse neurocognitive outcomes and its genotype is unrelated to indices of neurocognitive impairment (20, 21). This finding is also confirmed in our sample in which 6 and 5 patients with respective neurocognitive and movement impairment had different genotypes (20/25, 20/26, 20/27, and c.780dupT).

Behavior disorders were observed in 5 patients having 20/25, 20/26, and 20/27 genotypes. It seems that the psychological development of patients with CCHS is influenced by hypoxia rather than the genotype. In fact, patients diagnosed with hyperactivity and attention-deficit and mild to severe genotypes received late diagnosis of CCHS, resulting in increased exposure to hypoxia (22). Ocular impairment is well-described, and the high incidence of strabismus, pupillary abnormalities, and convergence insufficiency may be a result of a neurological defect in the midbrain (23). As already published, strabismus is the most frequent alteration in our patients in which 20/26, 20/27, and c.780dupT genotypes were detected.

In addition, the CCHS is characterized by (ANSD) (24). It has been verified that there is a relationship between the number of PARMS in PHOX2B gene and the phenotype of ANSD (4).

Affected patients can display several associated cardiovascular symptoms reflecting ANSD ranging from arrhythmia to lower and higher blood pressure, respectively, during awake and sleep periods. Gronli et al. (25) showed that patients with genotypes of 20/26 and 20/27 are at increased risk of prolonged asystole. This data is also confirmed in our study population, as 1 patient with atrio-ventricular block, requiring pacemaker implantation had 20/27 polyalanine repeat expansion. Hypoglycemia is another manifestation of ANSD. While the underlying mechanism of hypoglycemia in patients with CCHS has not well-been determined, there is an association with the severity of genotype. Six cases of patients affected with CCHS suffering from hypoglycemia associated with seizure are already published (26). In our case, recurrent hypoglycemia without hyperinsulinemia and with seizures was observed in two female children with c.780dupT and 20/27 mutations.

Breath-holding spells were also present in patients with c.780dupT, suggesting a more severe autonomic dysfunction and hence a more severe phenotype in NPARMs.

Since swallowing-induced peristalsis is centrally controlled and depends on the neural crest-derived esophageal innervation, it is not unlikely to find dysphagia in this population (27). In our sample, 6 patients needed placement of a gastrostomy tube due to abnormal swallowing. As observed in our patients having 20/26, 20/27, and c.780dupT mutations, the dysfunction of the central structures that control swallowing is probably related to the severe genotypes (27).

Finally, the tumor of neural crest origin was detected early in one child with c.780dupT mutation, confirming the greatest risk of developing this kind of tumor in patients affected with NPARMs (10).

Our study has some limitations as the retrospective design, the poor sample, and the heterogeneity of the described population composed of patients of different ages and who had experienced different primary care settings regarding the diagnosis and the ventilation support.

Nevertheless, we can confirm—based on our experience—that severe phenotype of CCHS is related to the number of polyalanine repetitions and NPARMs. Moreover, we observed that tracheostomy was most used in patients treated before 2010, and also in those affected with mild genotype/phenotype. After 2010, tracheostomy has been reserved, especially in patients with severe genotype/phenotype and the decannulation program has been carried out whenever possible (Figure 3). NIV has been considered as the first choice for ventilator support in patients with both mild and moderate to severe genotype/phenotype. None of our patients has been treated by negative pressure or diaphragm pacing.

Although guidelines for the diagnosis and management of CCHS have recently been published (28), we have so far adopted an internal clinical protocol that appears intensive above all in the 1st year of life. But both the growth in height and weight and the extreme variability in the amount of ventilator assistance required in this period of the life of the children, induced us to perform a quite frequent clinical and functional follow-up. Moreover, the difficult adaptation to ventilation support in 10 infants who often required the revision of ventilation setting, the need for nutritional adjustments in 2 patients with recurrent hypoglycemia, and the early diagnosis of neural crest tumor in one child, convinced us to draw up a clinical protocol based on the “precautionary principle,” that is the principle applied to the hypothetical risk to develop some known complications related to the disease that do not necessarily occur in all patients.

## Conclusions

Our study confirms that more severe phenotypes of CCHS are related to the number of polyalanine repetitions or to NPARMs. The novel heterozygous mutation c.780dupT on PHOX2B gene is associated with more ventilation dependency and autonomic dysfunction, predicting a more severe phenotype. The heterozygous mutation of C.225–256delCT on the same gene has a variable expression with atypical, mild, and late onset of phenotype in a female adult patient and classic, severe, and early onset of phenotype in a male child. The last observation reinforces the hypothesis that unknown cofactors could influence the phenotype and contribute to the variability of the clinical expression of CCHS.

Although invasive ventilation is often required by patients with severe genotype/phenotype, gradual acquisition of specific skills in the management of patients with CCHS, and technological improvements in mechanical ventilation, allowed us to improve our therapeutic approach in this population and to define a precise and scheduled follow-up program.

## Data Availability Statement

The original contributions presented in the study are included in the article/supplementary materials, further inquiries can be directed to the corresponding author/s.

## Ethics Statement

The studies involving human participants were reviewed and approved by Institutional Review Board. Written informed consent to participate in this study was provided by the participants' legal guardian/next of kin.

## Author Contributions

FP, MGP, and CC: conceptualization and writing—original draft preparation. AS and MBCT: review and editing. RC: supervision and editing. All authors contributed to the article and approved the submitted version.

## Conflict of Interest

The authors declare that the research was conducted in the absence of any commercial or financial relationships that could be construed as a potential conflict of interest.

## Abbreviations

CCHS: central congenital hypoventilation syndrome
PARMs: polyalanine repeat expansion mutations
NPARMs: non-polyalanine repeat expansion mutations
HSCR: Hirschsprung's disease
ANSD: autonomic nervous system dysfunction/dysregulation
LO–CCHS: late-onset central congenital hypoventilation syndrome
ICU: Intensive Care Unit
PICU: Pediatric Intermediate Care Unit
IMV: invasive mechanical ventilation
NIV: non-invasive ventilation
CNS: central nervous system
MRI: magnetic resonance imaging.
